# Disease-associated DNA methylation signatures in esophageal biopsies of children diagnosed with Eosinophilic Esophagitis

**DOI:** 10.1186/s13148-021-01072-y

**Published:** 2021-04-17

**Authors:** Caterina Strisciuglio, Felicity Payne, Komal Nayak, Marialuisa Andreozzi, Alessandra Vitale, Erasmo Miele, Matthias Zilbauer

**Affiliations:** 1 Department of Woman, Child and General and Specialistic Surgery, University “Vanvitelli”, via De Crecchio 4, CAP 80121 Napoli, Italy; 2 Department of Paediatrics, University of Cambridge, Level 8 Addenbrooke’s Hospital, Box 116, Hills Road, Cambridge, CB2 0QQ UK; 3 Department of Paediatric Gastroenterology, Hepatology and Nutrition, Cambridge University Hospitals, Addenbrooke’s, Cambridge, UK; 4 Department of Translational Medical Science, Section of Paediatrics, University of Naples “Federico II”, Naploli, Italy; 5 Wellcome Trust-Medical Research Council Stem Cell Institute, University of Cambridge, Cambridge, UK

**Keywords:** Eosinophilic Esophagitis, Epigenetics, DNA methylation, Mucosal biopsy

## Abstract

Eosinophilic esophagitis (EoE) is a leading cause of dysphagia and food impaction in children and adults. The diagnosis relies on histological examination of esophageal mucosal biopsies and requires the presence of > 15 eosinophils per high-powered field. Potential pitfalls include the impact of biopsy sectioning as well as regional variations of eosinophil density. We performed genome-wide DNA methylation analyses on 30 esophageal biopsies obtained from children diagnosed with EoE (*n* = 7) and matched controls (*n* = 13) at the time of diagnosis as well as following first-line treatment. Analyses revealed striking disease-associated differences in mucosal DNA methylation profiles in children diagnosed with EoE, highlighting the potential for these epigenetic signatures to be developed into clinically applicable biomarkers.

**Supplementary information:**

The online version contains supplementary material available at 10.1186/s13148-021-01072-y.

## Introduction

Eosinophilic esophagitis (EoE) is a chronic, allergic/immune-mediated inflammatory disease and the leading cause of dysphagia and food impaction in children as well as adults [1]. The condition and its symptoms have a major impact on patient health and quality of life, resulting in a substantial healthcare burden as the disease requires complex diagnostic and treatment approaches [2]. Diagnosis relies on the presence of symptoms suggestive of esophageal dysfunction and eosinophilic infiltration of the esophageal mucosa (i.e., the presence of ≥ 15 eosinophils per high-powered field, eos/hpf) [3]. Potential diagnostic pitfalls include the impact of biopsy sectioning, as well as regional variations of eosinophil density [4]. Furthermore, a reduction in eosinophils following treatment does not always coincide with improved clinical symptoms, suggesting that other mechanisms also contribute to disease pathogenesis [5]. Candidate gene and genome-wide association studies have identified several disease-associated susceptibility genes for EoE; however, given the rapid increase in incidence, as well as a wide variation of clinical phenotype and outcome, genetic variation is unlikely to be the sole contributing factor [6]. Increasingly, environmental factors and their potential impact on the human epigenome of disease relevant cell types are being considered [6]. A potentially promising epigenetic marker is DNA methylation. Operating in a highly cell type specific manner, this stable epigenetic marker has been used as a proxy to determine cell composition in mixed cell tissue samples, as well as in the development of clinical biomarkers [7–9]. Indeed, a recent study by Jensen and colleagues has highlighted distinct differences in the DNA methylation profile of esophageal biopsies in EoE patients according to treatment response [5]. Nevertheless, the potential role of epigenetic mechanisms in EoE disease pathogenesis and/or the value of DNA methylation signatures as clinical biomarkers remains poorly understood.

Here, we set out to perform genome-wide DNA methylation analyses on esophageal biopsies obtained from children with EoE and matched controls, with an aim to identify disease specific epigenetic signatures that could be of potential clinical value.

## Methods

### Patients

A total of 20 children aged between 4 and 16 years were prospectively recruited following informed consent. Ethical approval for this study was obtained from the Institutional Review Board of the University of Naples “Federico II” with the registration number 247/20. Diagnosis of Eosinophilic Esophagitis (EoE) was made according to current UEG guidelines [10]. Further details can be found in the Additional file 3: Supplementary Methods. In total, we obtained 30 esophageal biopsies from patients diagnosed with EoE (*n* = 7) and matched non-EoE/healthy controls (*n* = 13). Patients were sampled at the point of diagnosis (i.e., treatment naïve), and a further biopsy sample was obtained following completion of first-line treatment (8 weeks of treatment). All patients were measured for number of eosinophils per high-powered field (eos/hpf) at diagnosis prior to treatment (T0) and again on follow-up after treatment (T1).

### Sample processing and DNA methylation profiling

DNA was extracted from mucosal biopsies using the AllPrep MiniKit (Qiagen), according to the manufacturer’s instructions and bisulfite—converted using Zymo DNA methylation Gold kit (Zymo Research). Genome-wide DNA methylation was profiled using the Illumina EPIC BeadChip platform (Illumina, Cambridge, UK).

### Data pre-processing and quality control

A detailed description and references for all analyses can be found in the Additional file 3: Methods. In brief, raw intensity data were processed to extract beta values from IDAT files. Data were then normalized using internal control probes to correct for between-array technical variation, filtered to remove poor quality or potentially confounding probes and checked for batch effects. No batch effects were observed. Principal component (PC) analysis was performed on the filtered dataset, identifying one outlier which was removed from all downstream analyses. Correlation with clinical phenotype and array batch was measured using Kendall’s test statistic for continuous variables and ANOVA for categorical variables. Biological duplicate samples were taken from middle esophagus for 5 patients (2 controls, 1 patient at diagnosis and 2 patients after treatment), and each duplicate pair checked for correlation (R^2^ = 0.79 – 0.85, Additional file 1: Supplementary Fig. 1).

**Fig. 1:**
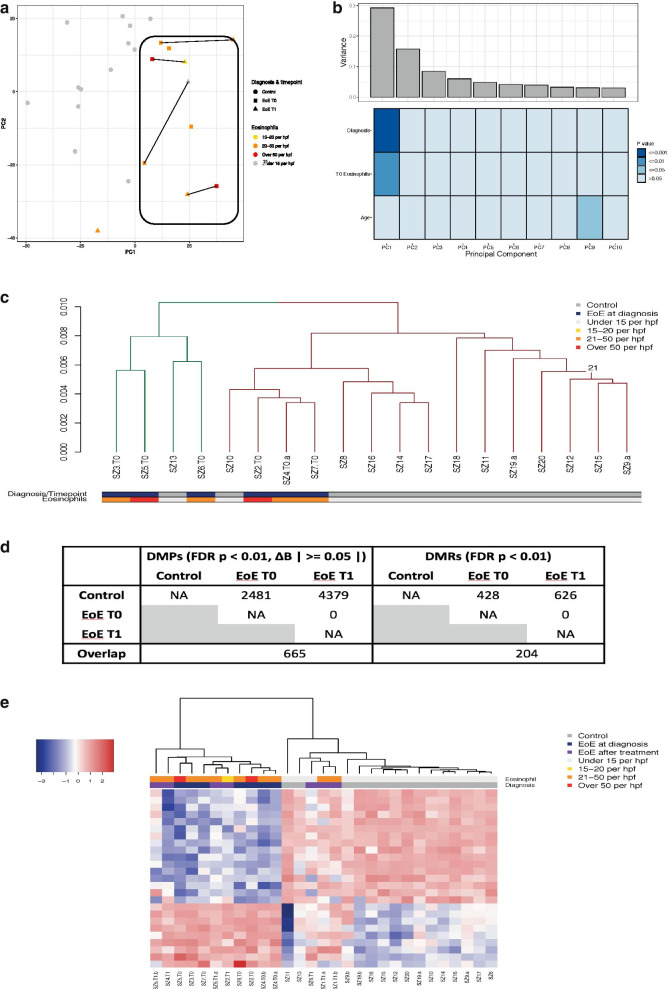
**a** Principal components plot (PC1, PC2) depicting patient diagnosis with number of eosinophils per high-powered field (eos/hpf) in controls (*n* = 13), and in EoE patients at diagnosis (EoE T0, *n* = 6) and after treatment (EoE T1, *n* = 5) after quality control. **b** Observed variance within all at diagnosis (T0) patient (*n* = 6) and non-EoE control samples (*n* = 13) in the first 10 principal components (top panel) against a heatmap showing the correlation between each principal component and phenotype, measured using Kendall’s test statistic for continuous variables and ANOVA for categorical variables (bottom panel). **c** Clustering of EoE patients at diagnosis (T0) and non-EoE controls (total *n* = 19) in all CpGs passing quality control using Pearson’s correlation with average clustering. The two principal clusters determined by hierarchical clustering are indicated in green and brown. **d** Summary of the significant differential methylation analysis results. To be considered as significantly differentially methylated, CpGs needed to have a False Discovery Rate (FDR) p-value < 0.01 and an absolute methylation difference (Δβ) ≥ 0.05. **e** Heatmap of all samples after quality control, excluding outliers but including biological duplicates (*n* = 29) subset for the top 25 CpGs significantly differentially methylated between EoE patients at diagnosis (T0) and non-EoE controls (FDR *p* < 0.01 and Δβ ≥|0.05|) using Pearson’s correlation with average clustering. DMP = Differentially methylated position, DMR = Differentially methylated region. EoE T0 = EoE patients at diagnosis, EoE T1 = EoE patients after first treatment

### Differential methylation

Duplicate samples were removed, and M-values calculated from the filtered beta values. Differential methylation analyses were then performed using the *limma* v3.42.2 [11] and *DMRcate* v2.0.7 [12] packages to detect differentially methylated positions (DMPs) and regions (DMRs), respectively, using a linear model with age and gender as covariates. To be considered as significantly differentially methylated, CpGs needed to have a False Discovery Rate (FDR) p-value < 0.01 and an absolute methylation difference (Δβ) > 0.05.

### Epigenetic clock

Normalised, filtered beta values were used to estimate epigenetic age using the Horvath online DNA Methylation Age Calculator [13] according to the recommended guidelines and resulting values compared to biological age.

## Results

Principal component analyses (PCA) of genome-wide DNA methylation profiles revealed a distinct separation of esophageal biopsies obtained from children newly diagnosed (i.e., treatment naïve) with EoE (*n* = 7) from healthy controls (*n* = 13, Fig. 1a). Interestingly, although DNA methylation signatures of biopsies obtained from EoE patients at a later stage of the disease (following first-line treatment) mostly displayed global changes (indicated by lines between samples Fig. 1a), the clear separation from normal controls remained. Next, we performed variance decomposition analyses in order to identify the main phenotypic factors contributing to observed variation in mucosal DNA methylation signatures. As shown in Fig. 1b, variation in DNA methylation profiles was significantly associated with diagnosis, number of eosinophils, as well as with age. Indeed, performing unsupervised clustering analysis further confirmed the presence of disease-associated DNA methylation signatures in patients diagnosed with EoE compared to healthy controls as clear clusters emerge that separate EoE patient from the majority of control samples (Fig. 1c).

In order to explore specific DNA methylation differences between patients with EoE and non-EoE controls that might form the basis for a potential diagnostic signature, we performed differential DNA methylation analyses. Given relatively low sample numbers, resulting in an inability to detect small methylation differences with confidence, we used a stringent threshold of significance, aiming to detect CpGs displaying a large effect size (false discovery rate, FDR, corrected p-value < 0.01 and an absolute methylation difference, Δβ, between groups of 0.05). This analysis yielded a total of *n* = 2,481 significant differentially methylated positions (DMPs) grouped into 428 differentially methylated regions (DMRs) between EoE patients and non-EoE controls at diagnosis and *n* = 4,379 DMPs (626 DMRs) between controls and EoE at follow-up (i.e., Time point T1). An overlap of *n* = 665 DMPs (204 DMRs) was identified between both comparisons, while analysis of EoE patients at diagnosis against EoE patients at T1 did not yield any significant DMPs even without filtering for results with Δβ ≥ 0.5 (Fig. 1d). Given that sample numbers for this final comparison are particularly low, it is possible that the lack of significant DMPs results from the increase in type II error expected in an experiment of this size and replication in larger datasets would be desirable, albeit beyond the scope of this study.

Based on previous reports of accelerated epigenetic age in the esophageal mucosa of EoE patients [5], we applied the Horvath online DNA Methylation Age Calculator [13] to estimate epigenetic age. Although these analyses confirmed a strong correlation with chronological age (R^2^ ≥ 0.8, *p* < 0.002, Additional file 2: Supplementary Fig. 2A & B), a comparison of EoE patients with non-EoE controls did not support the previously reported significantly disease-associated acceleration in epigenetic aging (p > 0.05 in our data) [5].

Finally, in order to evaluate the potential value of DNA methylation as a diagnostic biomarker in EoE, we selected the top 25 most significant DMPs resulting from the comparison between EoE patients at diagnosis and healthy controls (Additional file 4: Supplementary Table 2) to cluster all available samples. As shown in Fig. 1E, unsupervised clustering separated EoE samples from non-EoE controls with an accuracy of 0.90 (true positive rate = 0.79, false positive rate = 0). Correlation between the methylation signature and eosinophil count was also confirmed (*p* < 0.005).

## Discussion

DNA methylation has been shown in many studies to be a highly stable, and relatively easy to profile epigenetic mark, making it an ideal candidate for the development of clinically relevant biomarkers [8, 9]. Furthermore, DNA methylation signatures are highly cell type specific and have been used to deconvolute the composition of mixed cell tissue samples such as blood [7]. In our study, we observed disease-associated differences in genome-wide DNA methylation profiles of esophageal biopsies obtained from children diagnosed with EoE and matched non-EoE controls. Indeed, the top 25 differentially methylated CpG loci were sufficient to separate most samples accurately according to diagnosis, highlighting the potential use of such signatures as diagnostic biomarkers in EoE. Interestingly, following initiation of first-line treatment for EoE, and despite reduced number of eosinophils in the esophageal mucosa, DNA methylation profiles in follow-up samples continued to differ significantly from those of healthy controls. While our sample size was insufficient to test for a potential correlation with treatment response, the discrepancy between DNA methylation signatures and mucosal eosinophil count further highlights the major potential of this epigenetic mark in providing additional information to further guide treatment. Indeed, a recent study by Jensen et al. demonstrated that DNA methylation changes in esophageal biopsies obtained from adults with EoE before and after treatment seemed to correlate with clinical signs of treatment response [5]. The authors also observed accelerated aging in EoE patient-derived esophageal tissue samples but not in healthy controls. Although we were unable to confirm these findings in our sample cohort, we did observe a significant association between DNA methylation and age further highlighting the importance of considering age in future studies.

In summary, our study adds further evidence for the potential use of DNA methylation signatures as clinical biomarkers in EoE. Further studies in larger datasets are required to address specific value of this epigenetic signature in aiding diagnosis, predicting prognosis and/or monitor response to treatment.

## Supplementary information

**Additional file 1: Supplementary Figure 1**. Plots depicting the correlation between all duplicate samples.

**Additional file 2: Supplementary Figure 2**. Chronological age versus epigenetic (DNAm) age as calculated using the Horvath epigenetic clock [6] labelled by both disease status (A) and number of eosinophils per high powered field (eos/hpf).

**Additional file 3. Supplementary Methods and Supplementary Table 1**. Overview of patient characteristics.

**Additional file 4. Supplementary Table 2:** List of the top 25 differentially methylated CpGs between non-EoE healthy controls and EoE patients at diagnosis (T0).

## Data Availability

All microarray data have been deposited in ArrayExpress, accession number: E-MTAB-9824. All analysis code is available on GitHub [14].

## Abbreviations

DMP: Differentially methylated position
DMR: Differentially methylated region
EoE: Eosinophilic Esophagitis
Eos: Eosinophils
Hpf: High-powered field
PCA: Principal component analysis
PPI: Protein pump inhibitors
