# Supplementary figures and images for: Disease-associated DNA methylation signatures in esophageal biopsies of children diagnosed with Eosinophilic Esophagitis
Source: Clin Epigenetics. 2021 Apr 17;13:81. doi: 10.1186/s13148-021-01072-y (PMC8052828; doi:10.1186/s13148-021-01072-y)

# Supplementary Figure 1

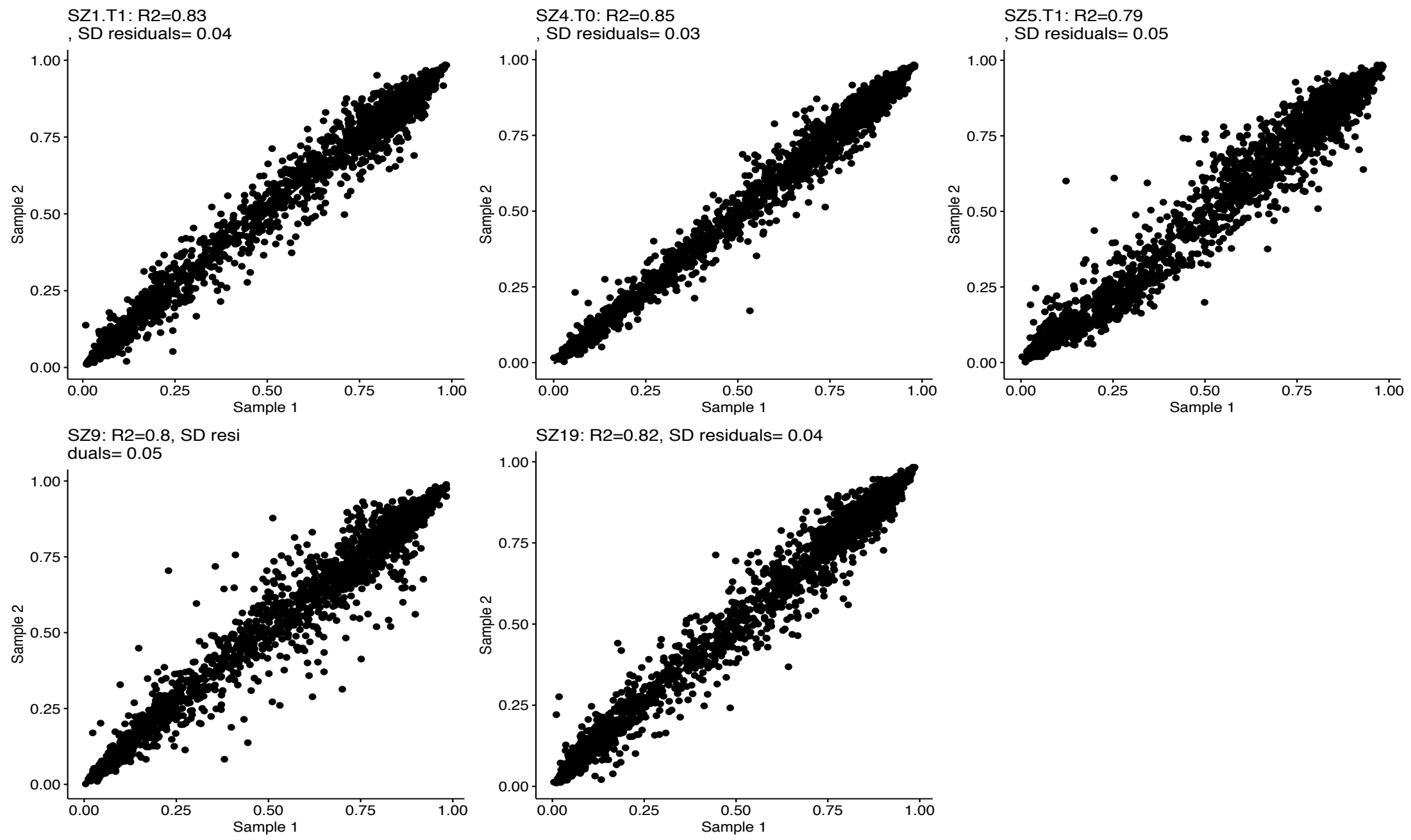

Supplement: Supplementary file 1 — Additional file 1: Supplementary Figure 1. Plots depicting the correlation between all duplicate samples. [file 13148_2021_1072_MOESM1_ESM.pdf]

# Supplementary Figure 2

A

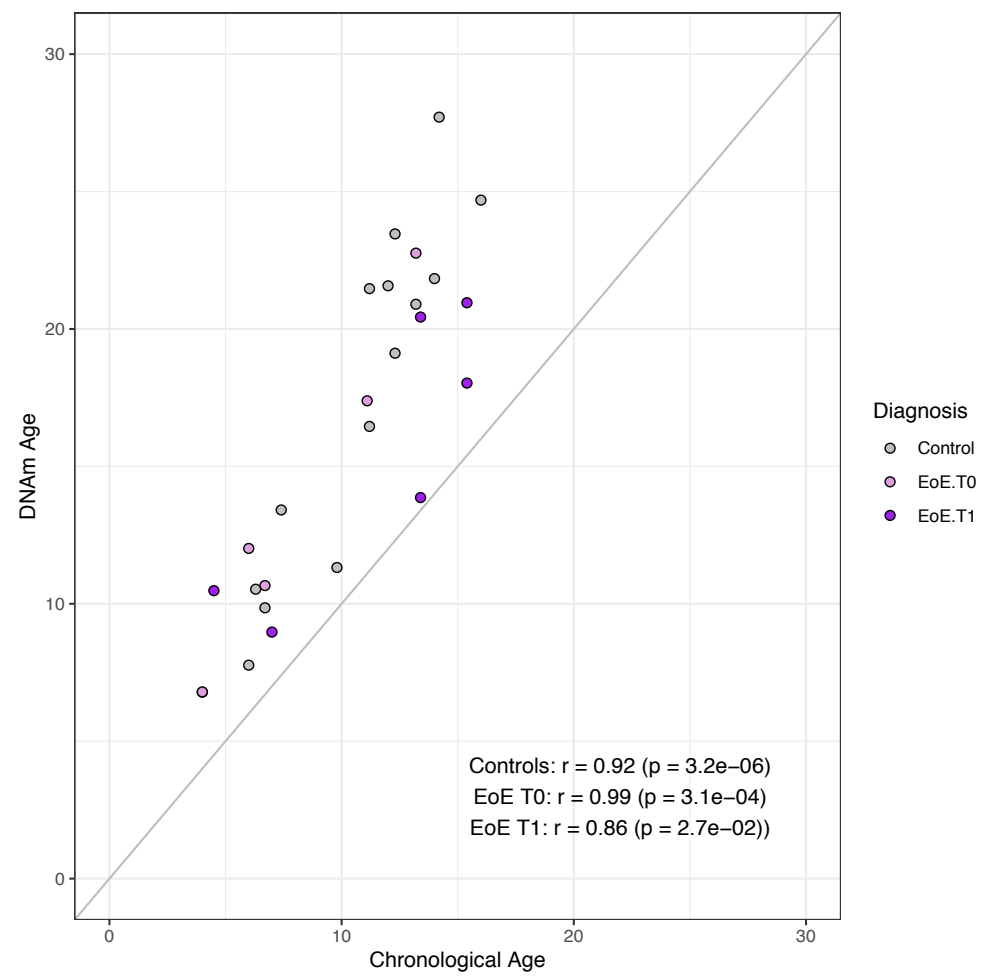

B

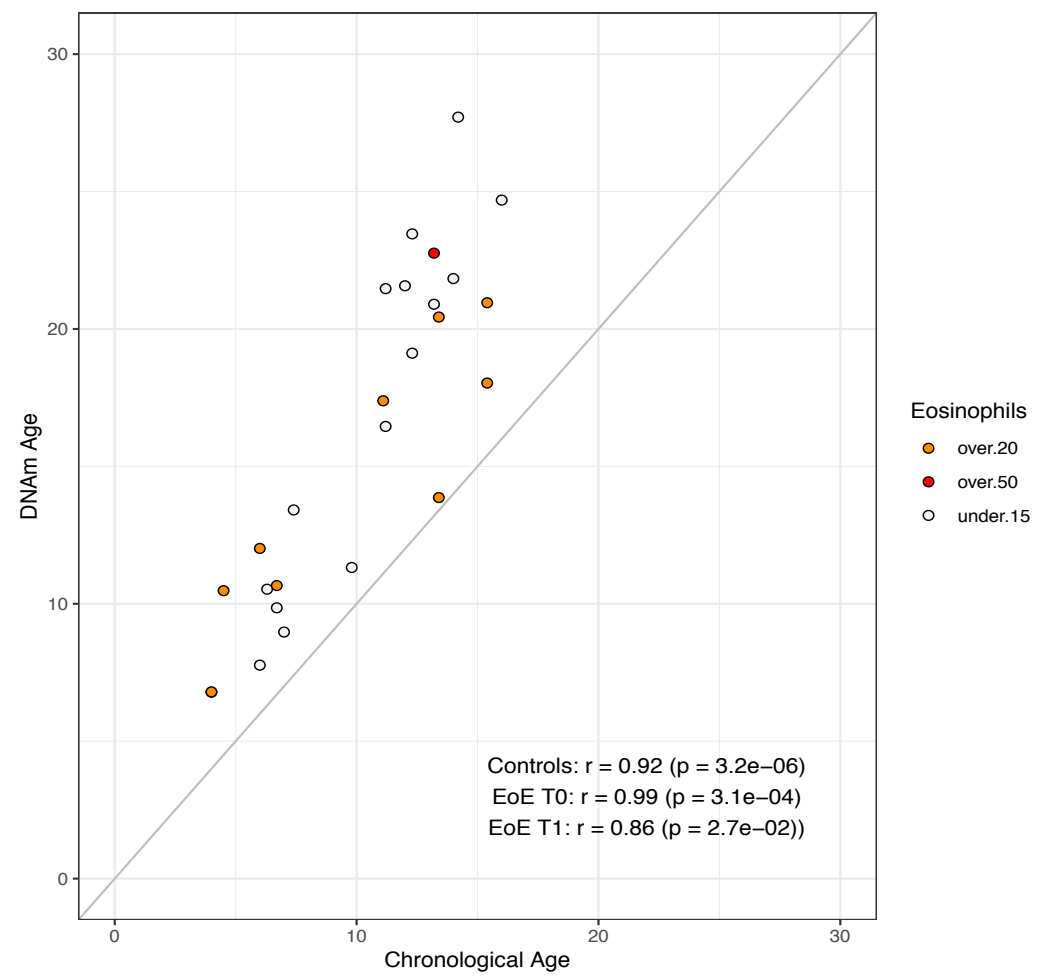

Supplement: Supplementary file 2 — Additional file 2: Supplementary Figure 2. Chronological age versus epigenetic (DNAm) age as calculated using the Horvath epigenetic clock [6] labelled by both disease status (A) and number of eosinophils per high powered field (eos/hpf). [file 13148_2021_1072_MOESM2_ESM.pdf]
