# Supplementary material for: Disease-associated DNA methylation signatures in esophageal biopsies of children diagnosed with Eosinophilic Esophagitis
Source: Clin Epigenetics. 2021 Apr 17;13:81. doi: 10.1186/s13148-021-01072-y (PMC8052828; doi:10.1186/s13148-021-01072-y)
Supplement: Supplementary file 3 — Additional file 3. Supplementary Methods and Supplementary Table 1. Overview of patient characteristics. [file 13148_2021_1072_MOESM3_ESM.docx]

**Ethical approval**

The Institutional Review Board of the University of Naples “Federico II” approved the study protocol with the registration number 247/20. Written informed consent was obtained from parents, and patients older than 13 years signed a statement of assent.

**Patients**

We conducted a case-control study of epigenetic methylation in esophageal tissue, utilizing prospectively collected esophageal biopsies and clinical data of a total 20 children aged between 4 and 16 years enrolled between January 2018 and June 2019, at the Department of Pediatrics, University of Naples “Federico II”. Diagnosis of Eosinophilic Esophagitis (EoE) was made according to consensus guidelines [1]. EoE patients were required to have at least one typical symptom of esophageal dysfunction, an esophageal biopsy demonstrating ≥15 eosinophils per high powered field (eos/hpf). Non-EoE controls were subjects with symptoms of esophageal dysfunction as noted above who, after endoscopy and biopsy, did not meet clinical and histologic criteria for EoE. In total, 7 patients were diagnosed with EoE and 13 as healthy controls.

All patients were measured for number of eosinophils (eos/hpf) at diagnosis prior to treatment (T0) and again on follow-up after treatment (T1) by an expereniced pathologist according to previously validated methodology [2]. The majority of patients (5/7) were treated for 8 weeks with proton pump inhibitor administered 1mg/kg per day. The remaining two patients received concomitant diagnosis of celiac disease and potential celiac disease and were started on a gluten free diet.

**Sample processing and DNA extraction**

During endoscopy, 1 or 2 biopsies from the middle oesophagus were obtained for research use and snap frozen. Additional clinical biopsies were taken from a different part of the oesophagus for EoE diagnosis.

DNA was extracted using the AllPrep MiniKit (Qiagen), according to the manufacturer’s instructions. DNA was quantified by spectrophotometry using a NanoDrop ND-1000 spectrophotometer. DNA was bisulfite- converted using Zymo DNA methylation Gold kit (Zymo Research).

**DNA methylation profiling**

Genome-wide DNA methylation was profiled on 200ng of DNA using the Illumina EPIC BeadChip platform (Illumina, Cambridge, UK), covering the whole genome at single CpG resolution (> 850,000 sites).

**Data pre-processing and quality control**

All analyses were performed in R version 3.6.3 and code is deposited in GitHub [3]. The raw intensity data was processed using the *minfi* package v1.28.4 [4] to extract beta values from IDAT files and detection p-values calculated to identify failed positions with reference to the background signal levels. Data were then normalised using the preprocessFunnorm method, which uses internal control probes to correct for between-array technical variation. Starting with a total of 864,859 probes mapping to the EPIC array, probes were filtered to remove those overlapping known genetic variants, present on the sex chromosomes, with potential to cross-hybridise to multiple genomic regions or of low quality as measured by a detection p-value > 0.05 in at least 1 % of the samples. After filtering, 780,380 CpGs remained for analysis. The filtered data was then checked for batch effects using the *BEclear* package v1.14.0 [5], with no batch effects detected (BEscore < 0.001 for all batches).

Principal component (PC) analysis was performed on the filtered dataset in order to identify outliers and to explore the main factors associated with variation in the dataset. One outlier sample was detected and removed from all downstream analysis. The first 10 PCs were examined for correlation with clinical phenotype and array batch using Kendall’s test statistic for continuous variables and ANOVA for categorical variables (Figure 1B and not shown). In addition, we investigated the distribution of sample variation with clinical phenotype more closely by plotting principal components (Figure 1A).

**Epigenetic clock analysis**

The Horvath epigenetic clock [6, 7] was used to estimate epigenetic age of all samples from normalised beta values, also implementing the clock specific normalisation function (Supplementary Figure 2). Epigenetic age was checked for correlation with chronological age and EoE patients were checked for accelerated epigenetic aging in comparison with the non-EoE controls.

**Duplicate analysis**

Biological duplicate samples were taken from middle oesophagus for 5 patients (2 controls, 1 patient at diagnosis and 2 patients after treatment). Each duplicate pair was checked for correlation in a random subset of 31,215 of the filtered probes (R^2^ = 0.79 – 0.85, Supplementary Figure 1).

**Differential methylation analysis**

Duplicate samples were removed, and M-values calculated from the filtered beta values. Differential methylation analyses were then performed using the *limma* v3.42.2 [8] and *DMRcate* v2.0.7 [9] packages to detect differentially methylated positions (DMPs) and regions (DMRs) respectively, using a linear model with age and gender as covariates. To be considered as significantly differentially methylated, CpGs needed to have a False Discovery Rate (FDR) p-value < 0.01 and an absolute methylation difference (delta beta) > 0.05. The top 25 most differentially expressed CpGs between non-EoE controls and EoE patients at diagnosis are listed in Supplementary Table 2. In order to determine the optimal number of clusters, the average silhouette width method was used with three different partitioning methods for validation (K-means, K-mediods and hierarchical). Two clusters were predicted as optimal by all methods.

**Availability of data and materials**

All microarray data have been deposited in ArrayExpress, accession number: E-MTAB-9824.

**Supplementary Table 1. Patient characteristics**

| **Number of patients** | **Cases**  **n = 7** | **Controls**  **n = 13** |
| --- | --- | --- |
| Median age (years) | 11 (4 – 15) | 12 (6 – 16) |
| Gender (% male) | 70 % | 70 % |
| Patients at diagnosis (duplicates) | 7 (1) | 13 (2) |
| Patients after treatment (duplicates) | 5 (2) |  |

1. Dellon ES, Liacouras CA, Molina-Infante J, Furuta GT, Spergel JM, Zevit N, Spechler SJ, Attwood SE, Straumann A, Aceves SS *et al*: **Updated International Consensus Diagnostic Criteria for Eosinophilic Esophagitis: Proceedings of the AGREE Conference**. *Gastroenterology* 2018, **155**(4):1022-1033 e1010.

2. Dellon ES, Fritchie KJ, Rubinas TC, Woosley JT, Shaheen NJ: **Inter- and intraobserver reliability and validation of a new method for determination of eosinophil counts in patients with esophageal eosinophilia**. *Dig Dis Sci* 2010, **55**(7):1940-1949.

3. **EoE Methylation Analysis** [<https://github.com/fp215/EOE_METHYLATION>]

4. Aryee MJ, Jaffe AE, Corrada-Bravo H, Ladd-Acosta C, Feinberg AP, Hansen KD, Irizarry RA: **Minfi: a flexible and comprehensive Bioconductor package for the analysis of Infinium DNA methylation microarrays**. *Bioinformatics* 2014, **30**(10):1363-1369.

5. Akulenko R, Merl M, Helms V: **BEclear: Batch Effect Detection and Adjustment in DNA Methylation Data**. *PLoS One* 2016, **11**(8):e0159921.

6. Horvath S: **DNA methylation age of human tissues and cell types**. *Genome Biol* 2013, **14**(10):R115.

7. McEwen LM, Jones MJ, Lin DTS, Edgar RD, Husquin LT, MacIsaac JL, Ramadori KE, Morin AM, Rider CF, Carlsten C *et al*: **Systematic evaluation of DNA methylation age estimation with common preprocessing methods and the Infinium MethylationEPIC BeadChip array**. *Clin Epigenetics* 2018, **10**(1):123.

8. Ritchie ME, Phipson B, Wu D, Hu Y, Law CW, Shi W, Smyth GK: **limma powers differential expression analyses for RNA-sequencing and microarray studies**. *Nucleic Acids Res* 2015, **43**(7):e47.

9. Peters TJ, Buckley MJ, Statham AL, Pidsley R, Samaras K, R VL, Clark SJ, Molloy PL: **De novo identification of differentially methylated regions in the human genome**. *Epigenetics Chromatin* 2015, **8**:6.

**SUPPLEMENTARY FIGURE AND TABLE LEGENDS**

**Supplementary Figure 1.** Plots depicting the correlation between all duplicate samples.

**Supplementary Figure 2.** Chronological age versus epigenetic (DNAm) age as calculated using the Horvath epigenetic clock [6] labelled by both disease status (A) and number of eosinophils per high powered field (eos/hpf).

**Supplementary Table 1.** Overview of patient characteristics

**Supplementary Table 2.** List of the top 25 differentially methylated CpGs between non-EoE healthy controls and EoE patients at diagnosis (T0).
